# TrkB Agonist Antibody Pretreatment Enhances Neuronal Survival and Long-Term Sensory Motor Function Following Hypoxic Ischemic Injury in Neonatal Rats

**DOI:** 10.1371/journal.pone.0088962

**Published:** 2014-02-14

**Authors:** Gab Seok Kim, Seongeun Cho, James W. Nelson, Gregory J. Zipfel, Byung Hee Han

**Affiliations:** 1 Department of Pharmacology, Seoul National University, College of Pharmacy, Seoul, Republic of Korea; 2 Wyeth Neuroscience Discovery Research, Princeton, New Jersey, United States of America; 3 Department of Neurological Surgery Washington University School of Medicine, St. Louis, Missouri, United States of America; 4 Hope Center for Neurological Disorders Washington University School of Medicine, St. Louis, Missouri, United States of America; 5 Department of Neurology, Washington University School of Medicine, St. Louis, Missouri, United States of America; Robert Debre Hospital, France

## Abstract

Perinatal hypoxic ischemia (H-I) causes brain damage and long-term neurological impairments, leading to motor dysfunctions and cerebral palsy. Many studies have demonstrated that the TrkB-ERK1/2 signaling pathway plays a key role in mediating the protective effect of brain-derived neurotrophic factor (BDNF) following perinatal H-I brain injury in experimental animals. In the present study, we explored the neuroprotective effects of the TrkB-specific agonist monoclonal antibody 29D7 on H-I brain injury in neonatal rats. First, we found that intracerebroventricular (icv) administration of 29D7 in normal P7 rats markedly increased the levels of phosphorylated ERK1/2 and phosphorylated AKT in neurons up to 24 h. Second, P7 rats received icv administration of 29D7 and subjected to H-I injury induced by unilateral carotid artery ligation and exposure to hypoxia (8% oxygen). We found that 29D7, to a similar extent to BDNF, significantly inhibited activation of caspase-3, a biochemical hallmark of apoptosis, following H-I injury. Third, we found that this 29D7-mediated neuroprotective action persisted at least up to 5 weeks post-H-I injury as assessed by brain tissue loss, implicating long-term neurotrophic effects rather than an acute delay of cell death. Moreover, the long-term neuroprotective effect of 29D7 was tightly correlated with sensorimotor functional recovery as assessed by a tape-removal test, while 29D7 did not significantly improve rotarod performance. Taken together, these findings demonstrate that pretreatment with the TrkB-selective agonist 29D7 significantly increases neuronal survival and behavioral recovery following neonatal hypoxic-ischemic brain injury.

## Introduction

Ischemic brain injury is a major cause of neurological impairments and mortality in adults and childhood, leading to severe cognitive and motor dysfunctions upon survival. Several key molecular and cellular processes responsible for ischemia-induced neuronal death have been identified in mature and developing brains, among which are glutamate and nitric oxide neurotoxicity, calcium accumulation, caspase activation and inflammatory activation [1], [2], [3], [4], [5]. Extensive biochemical and histochemical analyses have detected evidence for necrosis, apoptosis following ischemic injury [6], [7], [8], [9], [10], [11], suggesting a heterogeneous phenotypic nature of cell death. Currently there are no clinically effective treatments that provide robust neuroprotection and/or recovery.

Neurotrophins (NTs) are large secreted peptides that play important roles in neuronal survival, differentiation, and synaptic plasticity. Brain-derived neurotrophic factor (BDNF), the endogenous agonist for the tropomyosin-related kinase B (TrkB), has been shown to exert strong survival and neuroprotective effects in the central nervous system (CNS) and to facilitate post-injury recovery and activity-dependent synaptic plasticity [12], [13], [14], [15], [16]. BDNF binding to TrkB induces subsequent activation of downstream signaling cascades (the ERK1/2 and AKT pathways) responsible for cell survival. For example, in the primary neuronal cultures, BDNF protected hippocampal neurons against glutamate toxicity and glucose deprivation [17], [18] and rescued cerebellar granular neurons from apoptotic stimuli [19], [20]. This neurotrophic factor prevented neuronal cell death *in vivo* when given by infusion or virus-mediated delivery [21], [22], [23], [24], and attenuated behavioral deficits following spinal cord injury and ischemic stroke [25], [26], [27], [28]. Moreover, BDNF administration after spinal root avulsion prevented death of motor neurons, reversed cholinergic enzyme deficiency and induced axonal outgrowth of severely damaged axon terminals [29]. Despite these promising observations, however, BDNF failed to show any efficacy in clinical outcome measures in patients with amyotrophic lateral sclerosis [30], [31]. There are multiple factors that limit utilization of BDNF as a practical therapeutic reagent, including its poor bioavailability and short plasma half-life, and a lack of specificity for NT receptors. The latter is important in regard to the fact that BDNF also binds to the low-affinity p75 neurotrophin receptor (p75NTR), activation of which is responsible for neuronal cell death in many experimental settings [26], [32], [33]. Given these opposite effects of activation of TrkB and p75NTR on neurons, the molecular intervention for the specific activation of TrkB receptor, but not p75NTR, is desired to protect neuronal injury.

It has been reported that a novel TrkB-selective monoclonal antibody, 29D7, binds the extracellular domain of TrkB (but not p75NTR), activates its downstream signaling pathways, and promotes neuronal survival and neurite outgrowth in rodent primary neuronal cultures [34]. We, therefore, utilized this TrkB-selective antibody to explore its neuroprotective action in a neonatal model of hypoxic-ischemic brain injury. A rodent model of neonatal H-I initially described by Rice et al. [35] has been widely used to study the pathophysiology and therapeutic approaches implicated in neonatal H-I brain injury, a major contributor to newborn infant death and long-term neurological abnormalities. We have previously reported that BDNF treatment protects the brain against H-I-induced injury in neonatal rats [4], [36]. Using this model, we demonstrate here that the TrkB agonist antibody 29D7 activated the key TrkB downstream cascades (ERK1/2 and AKT), significantly reduced brain damage following H-I injury, and led to long-lasting functional improvements.

## Materials and Methods

### Materials

The TrkB-selective monoclonal antibody 29D7 and isotype-matching control IgG were generated as previously described [34] and provided by Wyeth Research. Recombinant human BDNF was purchased from Peprotech (Rocky Hill, NJ).

### Animals and the Surgical Procedure

All experimental protocols were approved by the Animal Studies Committee at Seoul National University. All surgery was performed under isoflurane anesthesia and all efforts were made to minimize suffering. Newborn Sprague-Dawley rats (both genders) were obtained from Samtako (Gyung-gi, Korea) when the pups were 3–4 days of age. Pups were housed with their dam in home cages under a 12/12 h light/dark cycle, with food and water freely available throughout the study. The neonatal H-I brain injury model was performed as previously described [35], [36], [37], [38] with modifications. At post-natal day 7 (P7), rats were anesthetized with 2.5% isoflurane and the left common carotid artery was permanently ligated. The incision was sutured and the pups were returned to the mother for a 2 h recovery and feeding period. Pups were then placed in a humidified hypoxic chamber with 8% oxygen (balance nitrogen) flow at 37°C for 2.5 h. Following H-I injury, pups were returned to their cages and remained with their mother.

### Experimental Groups

**Figure 1:**
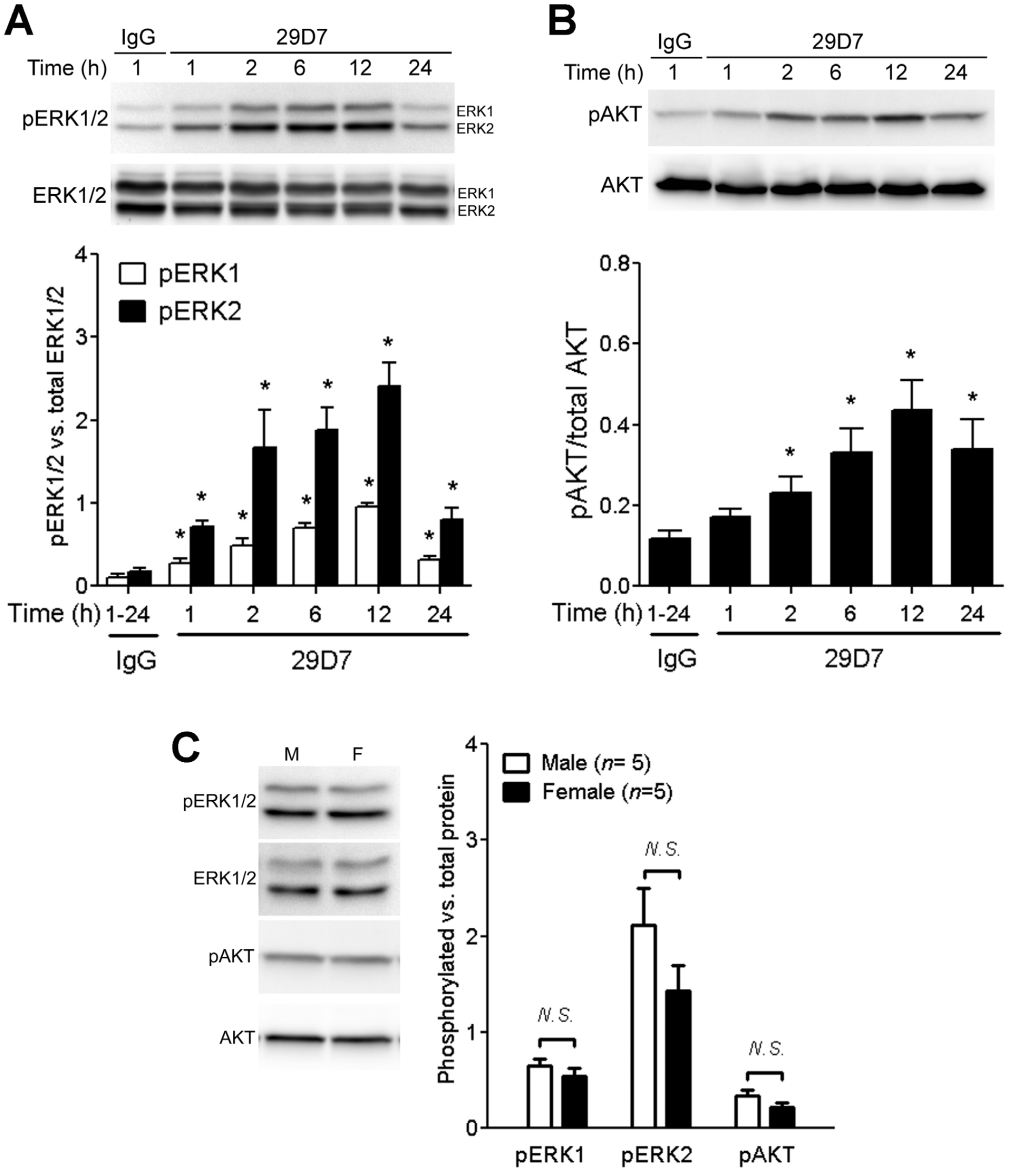
29D7 increases phosphorylation of ERK1/2 and AKT in vivo. P7 rat pups received an icv injection of control IgG or 29D7 (0.3 nmol in 5 µl PBS) and the cortical tissues were dissected at various time points as indicated. Following preparation of tissue lysates, proteins (30 µg/lane) were separated by SDS-PAGE and transferred to nitrocellulose membranes. Immunoblotting was performed with antibodies specific to phosphorylated ERK1/2 (pERK1/2) and total ERK1/2, and phosphorylated AKT (pAKT) and total AKT proteins. Signal intensity was quantified by densitometry and normalized to corresponding total proteins. Since IgG treatment had no effect on the levels of pERK1/2 and pAKT (*P*>0.05) for up to 24 h, all time points of the IgG-treated groups were pooled. Data represent mean ± SEM (*n* = 4–5). *P<0.05 compared with the IgG-treated group by ANOVA followed by Dunnett’s multiple comparison (A–B). **C.** Levels of phosphorylated ERK1/2 and phosphorylated AKT were compared in male vs. female mice 2–6 h after icv injection of 29D7, 0.3 nmol (*n* = 5). N.S.: no significance between male and female mice assessed by *t*-test (*P*>0.05).

The first set of experiments was performed to assess whether 29D7 induces activation of ERK1/2 and AKT in control (no H-I surgery) P7 rats. Two groups of rats were used: 1) control IgG, 0.3 nmol (*n* = 10) and 2) 29D7, 0.3 nmol (*n* = 50). At various time points indicated in Fig. 1 after the injection, cortical brain samples (*n* = 5 per time point) were prepared for Western blot and immunohistochemical analyses. The second set of experiments was designed to examine the effect of the TrkB agonist on apoptosis in the brain 24 h following H-I. Three groups of P7 rats were used: 1) IgG (*n* = 12), 2) BDNF (*n* = 10), and 3) 29D7 (*n* = 12). The third set of experiments was designed to determine the effect of 29D7 on brain tissue loss 7 days following H-I. Three groups of rats were used: 1) control IgG (*n* = 25), 29D7, 0.1 nmol (*n* = 15), and 29D7, 0.3 nmol (*n* = 15). The fourth set of experiments was designed to assess the effect of 29D7 on body temperature. Three groups of P7 rats were used: 1) sham surgery control (*n* = 6), 2) H-I:IgG (*n* = 7) and 3) H-I:29D7 (*n* = 7). The fifth set of experiments was designed to examine long-term behavioral outcome and brain tissue loss following H-I. Three groups of rats were used: 1) sham surgery:IgG (*n* = 6), 2) H-I:IgG (*n* = 13), and 3) H-I:29D7 (*n* = 13). We found that mortality was not significantly different between IgG- and 29D7-treated groups when assessed 24 h post H-I (IgG: 11.8% vs. 29D7: 12.2%, P>0.05). All assessments were performed by investigators blinded to the surgical procedure and treatments.

### Intracerebroventricular (icv) Administration

For drug treatments, pups received icv administration of BDNF, 29D7, or isotype-matching control antibody (IgG) just prior to hypoxic injury into the left ventricle ipsilateral to H-I injury per our published protocol [36], [39]. Under isoflurane anesthesia, rats were subjected to icv injection (2 mm rostral to bregma, 1.5 mm lateral, and 2 mm deep to the skull surface) in a volume of 5 µl using a Hamilton syringe with a 27 gauge needle.

### Measurement of Body Temperature

Body temperature was monitored using a digital infrared ear thermometer (Black & Decker, Pasadena, CA). Body temperature was firstly recorded and animals immediately received an icv administration of 29D7 or control IgG followed by exposure to hypoxia for 2.5 h. The second measurement of body temperature was performed after completion of hypoxia.Pups were then returned to their dam andbody temperature was measured up to 7 days post-H-I.

### Western Blot Analysis

Brain tissues from the cortex were dissected and frozen in dry ice. Tissue samples were homogenized in a lysis buffer (10 m***m*** HEPES, pH 7.4, 5 m***m*** MgCl_2_, 1 m***m*** dithiothreitol, 1% Triton X-100, 2 m***m*** EGTA, 2 m***m*** EDTA, 1 m***m*** phenylmethylsulfonyl fluoride and Protease Inhibitor Cocktail) and centrifuged at 12,000×g for 10 min at 4°C. Proteins (30 µg/lane) were separated by sodium dodecyl sulfate–polyacrylamide gel electrophoresis and transferred to nitrocellulose membranes (Bio-Rad Laboratories, Hercules, CA, USA) as previously described [4], [36]. Blots were blocked with 3% dried milk in Tris-buffered saline containing 0.05% Tween 20 at 25°C for 2 h. Blots were then incubated with primary antibodies, followed by incubation with anti-rabbit or anti-mouse horseradish peroxidase-conjugated IgG and visualized with enhanced chemiluminescence (LabFrontier, Seoul, Korea). Primary antibodies used were as follows: rabbit anti-caspase 3 (1∶1000, Cell Signaling Technology, Danvers, MA); rabbit anti-α-spectrin (1∶2000, Chemicon, Temecula, CA); anti-β-actin (1∶3000); and anti-poly(ADP-ribose) polymerase (PARP) (1∶1000, Santa Cruz Biotechnology, Santa Cruz, CA), and anti-phospho-ERK1/2 (1∶1000), anti-phospho-AKT (1∶1000), anti-total EKK1/2 (1∶2000) and anti-total AKT (1∶2000) antibodies from Cell Signaling Technology.

### Caspase-3 Activity Assay

Twenty-four hours after H-I, the hippocampal and cortical tissues from both lesioned and unlesioned hemispheres were rapidly dissected and Asp-Glu-Val-Asp-(7-amino-4-methylcoumarin) (DEVD-AMC) cleavage activities were measured as previously reported [4], [36]. Tissue lysates (10 µl) were incubated with 90 µl of an assay buffer (10 mM HEPES, pH 7.4, 42 mM KCl, 5 mM MgCl_2_, 1 mM DTT, and 10% sucrose) containing 30 µM acetyl-DEVD-AMC (Calbiochem, San Diego, CA) and the enzymatic activities were calculated by kinetic analyses of the emitted fluorescence (ex: 360 nm; em: 460 nm) measured every 5 min for 30 min. Acetyl-AMC (Calbiochem) was used to obtain a standard curve and the enzyme activity was presented as picomoles of AMC per milligram of protein per minute.

### Assessment of Brain Damage following Hypoxic-ischemic Injury

Regional brain loss was determined at 7d or 35d post H-I injury by calculating the amount of surviving tissue in coronal sections as previously described [36], [40]. Briefly, coronal sections from the genu of the corpus callosum to the end of the dorsal hippocampus were stained with cresyl violet. The cross-sectional areas of the striatum, cortex and hippocampus in each of eight equally spaced reference planes were photo-scanned and the area of each brain region was calculated using Adobe Photoshop 6.0. The sections utilized for quantification in a blinded manner corresponded approximately to Plates 20, 25, 30, 35, 51, 56, 61 and 66 in the rat brain atlas of Paxinos and Watson [41].

### Immunofluorescent Labeling and Microscopy

Brain tissues were prepared as described previously [36], [42], [43] and subjected to immunofluorescent labeling. Briefly, tissue sections were blocked with Tris-buffered saline containing 1% bovine serum albumin, 0.2% dry milk and 0.3% Triton X-100 for 30 min and incubated overnight with primary antibodies, followed by incubation for 2 h with fluorescence-labeled secondary antibody Alexa-488 or Alexa-568 (Invitrogen, Carlsbad, CA). After incubation with fluorescence-labeled secondary antibody, slides were cover-slipped with Vectashield mounting media (Vector Laboratories, Burlingame, CA) and examined with an IX71 fluorescence microscope (Olympus, Hamburg, Germany) or a Zeiss LSM confocal microscope (Zeiss, Oberkochen, Germany). The following primary antibodies were used: anti-NeuN antibody conjugated with Alexa 488 (1∶100; Millipore, Billerica, MA), and anti-phospho-ERK1/2 (1∶1,000, Cell Signaling Technology).

### Tape-removal Test

Sensorimotor dysfunction was assessed by a tape removal test as described [44], [45] in a blind manner. Animals were pre-trained for 3 days before tape-removal test on P28, P35, and P42 (3–5 weeks after H-I injury). Round sticky labels 12-mm in diameter were applied to the animal’s forelimbs bilaterally and the latency time required to remove the labels from forelimbs was recorded. The maximum time allowed to remove the labels was 60 sec. Three trials per animal were performed and averaged.

### Rotarod Test

We performed a rotarod test to examine motor coordination per the published protocol [46] with modification. Rats were placed on the rota-rod treadmill (Rotamex-5, Columbus Instrument, Columbus, OH), which has an accelerating motor-driven treadmill and the rotation speed was accelerated from 4 to 40 rpm over 180s. Rats performed two trials with a 15 min inter-trial interval at postnatal days 28, 29 and 30.

### Statistical Analyses

Data were expressed as means ± SEM. Comparison among multiple groups were performed with a one-way analysis of variance (ANOVA) followed by Dunnett’s multiple comparison method. *P* value <0.05 was considered significant.

## Results

### 29D7 Activates ERK1/2 and AKT in Neurons of the Neonatal Brain

**Figure 2:**
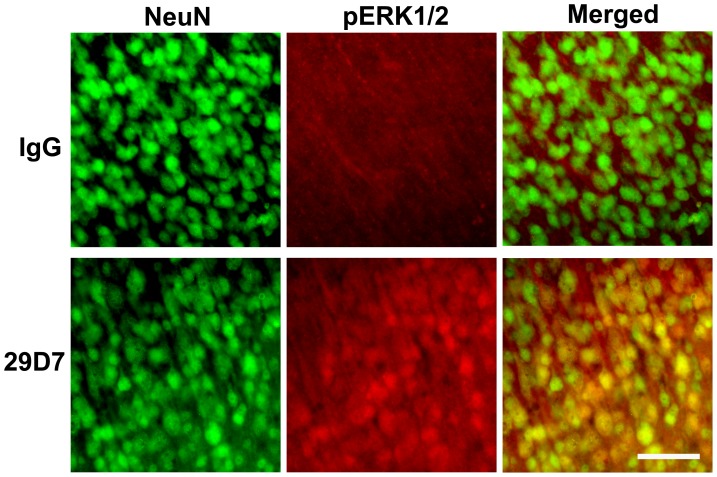
29D7 induces phosphorylation of ERK1/2 in neurons. P7 rats received an icv injection of either IgG or 0.3(*n* = 5 per group). Coronally sectioned brain sections were immunofluorescently double-labeled with anti-phospho-ERK1/2 (pERK1/2) antibody (red) and a neuron-specific marker, NeuN (green), followed by fluorescent microscopy. Note that pERK1/2 signals are co-localized with NeuN staining (indicated by yellow signals) in the cortex ipsilateral to icv injection. Scale bar: 50 µm.

Previous studies have demonstrated that endogenous TrkB agonists such as BDNF can activate both the ERK1/2 and PI3K-AKT pathways in responsive cells [47], [48]. We first asked whether the TrkB agonist antibody 29D7 could activate these pathways in the neonatal brain *in vivo*. Western blot analyses revealed that icv injection of 29D7 (0.3 nmol) increased ERK1/2 phosphorylation and AKT phosphorylation in the cortex ipsilateral to the icv injection (Fig. 1). Levels of both phosphorylated ERK1 and phosphorylated ERK2 were significantly increased by 1 h and lasted up to 24 h after the injection (Fig. 1A). Similarly, 29D7 significantly increased ATK phosphorylation at least for up to 24 h. We also found that increased phosphorylation of ERK1/2 and AKT was noted in the hemisphere contralateral to the injection, indicating that the antibody could reach to the entire brain after icv injection (data not shown). However, icv injection of control IgG (0.3 nmol) was no effect on the levels of phosphorylated ERK1/2 and phosphorylated AKT for up to 24 h (P>0.05, data not shown). Based on the fact that TrkB is expressed in neurons in the brain [49], [50], we hypothesized that activation of ERK1/2 and AKT by 29D7 may primarily occur in neurons. To address this, brain tissues were prepared at various timepoints after icv injection of 29D7 and subjected to immunohistochemical analyses. Similarly to our previous report [36], phosphorylated ERK1/2 (pERK1/2)-immunoreactivity (−IR) was detectable mostly in neuronal cell processes in the IgG-treated P7 control rats with no obvious staining in the cell bodies or nuclei. However, upon icv injection of 29D7, pERK1/2-IR was greatly increased in all cellular compartments, including neuronal processes, cell bodies, and nuclei in neuronal layers of the cortex and the hippocampus (Fig. 2). The prolonged increase in the pERK1/2-IR was noted in neuronal cell processes through all layers in the cortex 24 h after the injection (data not shown). Double labeling with a neuron-specific marker, NeuN, revealed that pERK1/2-IR was localized to neurons (Fig. 2).

### 29D7 Reduces Acute Apoptotic Brain Damage following Hypoxic-ischemic Injury

**Figure 3:**
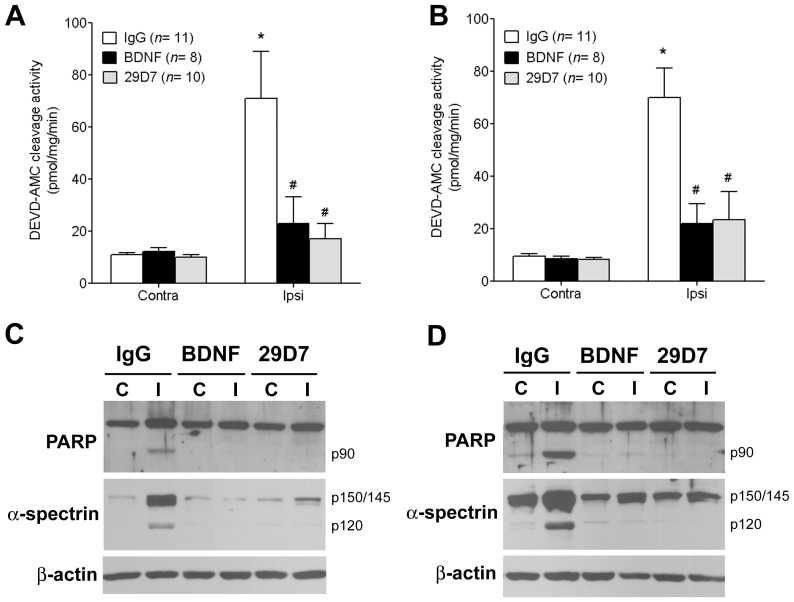
29D7 blocks caspase-3 activation following H-I brain injury. P7 rats underwent left carotid ligation followed by hypoxic injury for 2.5-four hours later, brain tissues were dissected from the hippocampus (A, C) and the cortex (B, D). **A, B.** Tissues were lysed and caspase-3 activity was determined by Asp-Glu-Val-Asp-7-amino-4-methyl-coumarin (DEVD-AMC) cleavage assay. Data indicate mean ± SEM. * p<0.05 compared with contralateral hemisphere; # p<0.05 compared with a IgG-treated ipsilateral hemisphere, as analyzed by ANOVA followed by Dunnett’s comparison method. **C, D**. Tissue proteins (30 µg/lane) were separated by SDS-PAGE and subjected to immunoblotting with antibodies specific to poly(ADP-ribose) polymerase (PARP), α-spectrin, and β-actin. Data shown are representative of 6 independent experiments.

BDNF, one of endogenous agonists for TrkB, has been shown to be neuroprotective against H-I injury in the developing rat brains via activation of the ERK1/2 pathways [36], [40]. We have reported that TrkB agonist antibodies elicit the stimulation of signaling cascades downstream of TrkB activation, resulting in promotion of neuronal survival and neurite outgrowth in culture with an equipotent efficacy to BDNF [34]. Therefore, we sought to further test if 29D7 has neuroprotective effects on neonatal H-I brain injury *in vivo*. Consistent with a previous study [39], H-I insult markedly increased the caspase-3 activity by 24 h (7–8 fold) in the hippocampus and the cortex of ipsilateral, but not contralateral, sides of the carotid ligation, as determined by DEVD-AMC cleavage assay (Fig. 3A, B). ICV administration of 0.3 nmol 29D7 or BDNF potently attenuated the H-I-induced caspase-3 activation compared to the IgG-treated group (*P*<0.05). In subsequent studies employing Western blot analysis, we also demonstrated that H-I resulted in an increase in the appearance of caspase-3-cleaved products of p90 poly (ADP-ribose) polymerase (PARP) as well as p120 α-spectrin 24 h post H-I in the hippocampus and the cortex (Fig. 3C,D). Treatments with 29D7 or BDNF blocked the proteolytic cleavages of both caspase-3 substrates, indicating their abilities to block caspase-3-dependent apoptosis following H-I injury (Fig. 3C, D).

### 29D7 Results in the Long-term Neuroprotection against H-I Injury

**Figure 4:**
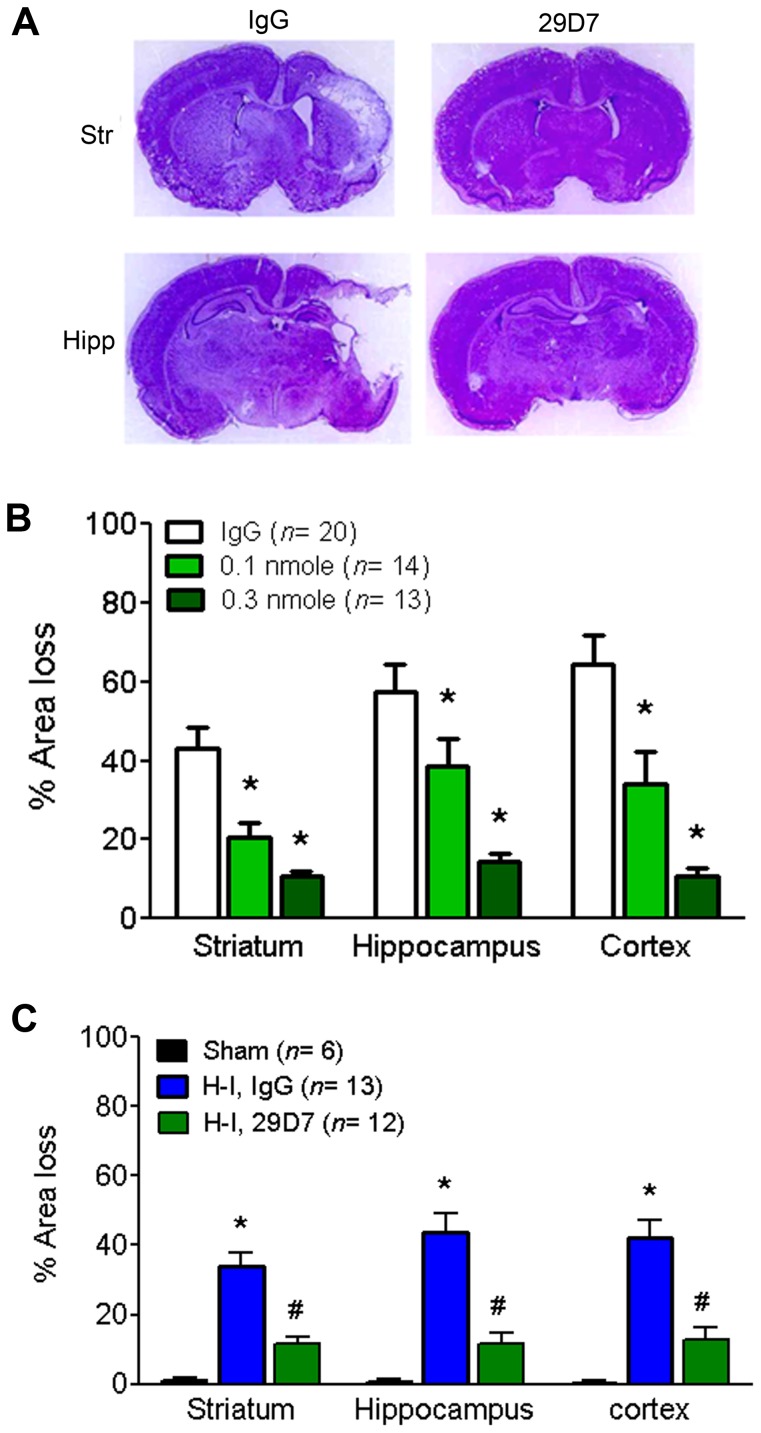
29D7 exerts long-term neuroprotection against H-I brain injury. P7 rats underwent left carotid ligation and hypoxia for 2.5(0.1 nmol or 0.3 nmol) prior to hypoxia. Seven days later, brains were removed, coronally sectioned, and stained with cresyl violet. **A.** Representative photographs illustrating hemispheric tissue loss after H-I injury. **B.** Area tissue loss from the striatum, hippocampus, and cortex was determined by comparing the area of surviving tissue with unlesioned (right) hemisphere. Data indicate mean ± SEM. *P<0.05 compared to the IgG-treated group. **C.** Animals used in behavioral studies in Fig. 5 were subjected to tissue loss analysis at P42. *P<0.05 compared to the sham-operated group; #P<0.05 compared to a control IgG-treated, H-I group.

**Figure 5:**
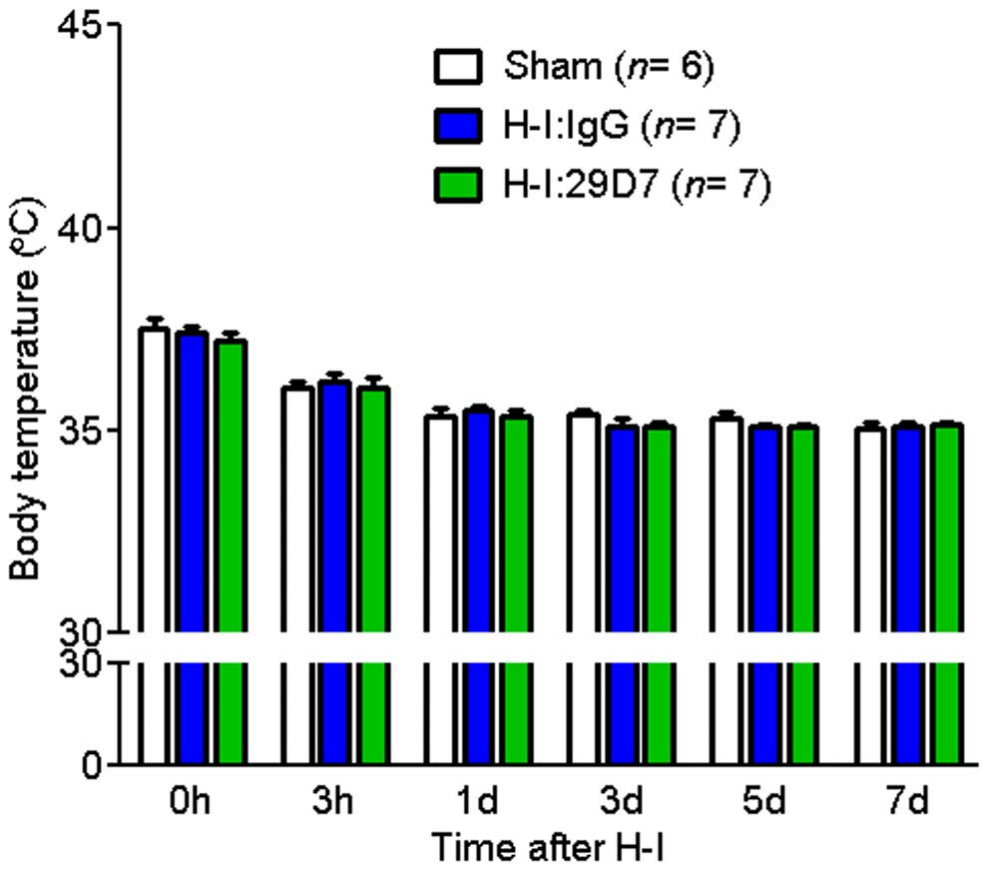
29D7 does not affect body temperature. P7 rats underwent left common carotid ligation or sham surgery, followed by hypoxia for 2.5-I injury were treated with an icv administration of control IgG or 29D7 immediately before hypoxia. Body temperature of the pups was measured at various time points indicated using a digital infrared thermometer. Data indicate mean ± SEM. No significance between groups assessed by one way ANOVA (*P*>0.05).

We explored long-term neuroprotective effects of 29D7 on H-I brain injury. Neonatal rats underwent H-I brain injury at P7 and hemispheric tissue loss was assessed 7 days after H-I. Consistent with previous reports [36], [39], an unilateral carotid ligation followed by exposure to hypoxia for 2.5 h resulted in brain tissue loss by 40–60% in the striatum, hippocampus, and cortex in the IgG-treated group compared to the unlesioned hemisphere (Fig. 4A–D). ICV injection of 29D7 (0.1 or 0.3 nmol) significantly reduced the brain tissue loss at 1 week in all brain regions studies in a dose-dependent fashion as compared to the IgG-treated group (Fig. 4A–B). This robust neuroprotective effect of 29D7 was also observed when the tissue loss was analyzed 5 weeks post H-I (Fig. 4C). To examine the possibility that neuroprotective effect of 29D7 was attributed to hypothermia which is shown to attenuate H-I brain injury [51], [52], body temperatures were monitored (Fig. 5). We found the body temperature of rats treated with 29D7 did not differ from those treated with control IgG or sham surgery for up to 7 days after H-I injury (P>0.05).

### 29D7 Prevents Long-term Neurological Dysfunction following H-I Brain Injury

**Figure 6:**
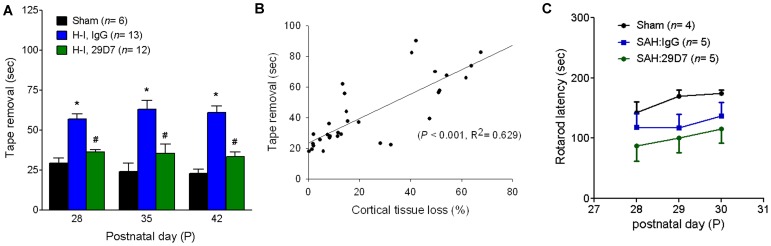
29D7 improves functional outcome following H-I injury. P7 rats underwent sham operation (Sham) or H-I brain injury by left carotid ligation and subsequent exposure to hypoxia for 2.5 h. H-I animals received an icv administration of 0.3 nmol control IgG or 29D7, 0.3 nmol prior to hypoxia. **A.** Sensorimotor function was assessed by a tape-removal test at P28, P35, and P42. Data represent mean ± SEM. *P<0.05 compared with sham; #P<0.05 compared with IgG treated group, analyzed by ANOVA and Dunnett’s multiple comparison test. **B.** Correlation between % cortical tissue loss and functional performances. Functional performance of individual animals in A was plotted against the percentage of cortical tissue loss, as exemplified in Figure 4C. **C.** Motor coordination function was assessed by a rotarod test 3 weeks after H-I injury. *P*>0.05 between groups.

We next sought to explore if 29D7-mediated neuroprotection was associated with long-term behavioral improvements by employing the sticky tape-removal method, a widely used test of somatosensory function in rodents [44], [45]. As shown in Fig. 6A, treatment with 29D7 significantly improved neurological dysfunctions as compared to the IgG-treated group (P<0.05) when assessed at P28–42 (3, 4, and 5 weeks after injury). Histological analysis of the brain tissue confirmed persistent neuroprotection by 29D7 at P42 (Fig. 4C). Performances in the tape-removal test significantly correlated with the cortical tissue loss (Fig. 6B, R ^2^ = 0.629, P<0.001). We performed a rotarod test to determine the effects of 29D7 on motor coordination and balance 3 weeks after H-I injury. Though there was a trend towards worse performance in both H-I:IgG and H-I;29D7 groups compared with sham-operated group, 29D7 did not appear to improve rotarod performance (*P*>0.05 bewteen H-I:IgG vs. H-I:29D7).

## Discussion

Our study here presents evidence that 1) the TrkB-selective agonist antibody 29D7 effectively activates the TrkB signaling cascades *in vivo*, 2) significantly inhibits neuronal cell loss, and 3) elicits long-term neuroanatomical and behavioral benefits against H-I injury. Cell survival-promoting effects of neurotrophins, in particular BDNF, are elicited by activation of different intracellular signaling cascades including the phosphatidylinositol 3-kinase (PI3-K) and AKT, and the extracellular signal-related kinase1/2 (ERK1/2) pathways [48], [53], [54]. We found that icv administration of 29D7 markedly increased levels of phosphorylation of both ERK1/2 and AKT proteins in the normal mouse brain. Double immunolabeling studies confirmed that ERK1/2 phosphorylation occurred in the neuronal population. Interestingly, 29D7-induced ERK1/2 phosphorylation in the cortex was slower in initiation compared to that in the hippocampus (data not shown), while the activity was long lasting at least up to 24 h. This regional difference might be attributed in part to temporal differences in access to the antibody between two tissues related to penetration distances. Differential kinetics of ERK1/2 activation has also been reported; however, a continuous exposure to BDNF over 2–6 h was required for a persistent phosphorylation of ERK1/2 for cortical neurons in culture [55], [56], while BDNF elicited a rapid and transient activation of ERK1/2 in hippocampal slices [57]. In fact, ERK1/2 activation in the hippocampus was previously shown to be more robust compared to other brain regions including the cortex, suggesting region-specific regulations of ERK1/2 activity in the brain [58].

Our results strongly demonstrated that pretreatment with 29D7 provided neuroprotection against H-I brain injury in P7 rats, as assessed by acute caspase-3 activation 24 h post-H-I as well as by long-term brain tissue loss 1–5 weeks following H-I insult (Figs. 3 and 4). Previous reports demonstrate that H-I insult to the developing brain results in mainly apoptotic cell death, accompanying typical hallmarks such as DNA laddering, nuclear condensation and delayed caspase-3 activation [4], [40]. Han and Holtzman found that icv administration of BDNF markedly protected the neonatal brain following H-I [36]. This BDNF’s neuroprotective action was blocked by pretreatment with an ERK1/2 inhibitor, U0126, but not with an PI3-K inhibitor, wortmannin [36], suggesting a central role of the ERK1/2 pathway in mediating the neuroprotecive effect of BDNF on H-I brain injury in developing brain. The notion that 29D7 robustly induces phosphorylation of ERK1/2 *in vivo* (Figs. 1 and 2) as well as *in vitro* [34] supports the idea that 29D7-mediated neuroprotection would be, at least in part, mediated via the ERK1/2-dependent mechanism. Further insights into the ERK1/2 downstream mediators that are required for protection against injury *in vivo* seem warranted to attempt to elucidate the molecular mechanism underlying neuroprotective action of 29D7 [59], [60].

In the present study, we found that the neuronal rescue was closely linked to a long-term improvement of somatosensory function (Fig. 6) as assessed by tape removal test, one of the most sensitive testes for detecting sensorimotor deficits [61] [44], [45]. For example, Yager et al. have compared various neurobehavioral tests following ischemic brain damage to establish correlations between functional performance and morphologic damage levels in rats of various age groups [46]. Interestingly, among the battery of tests conducted in their study, including rotarod, foot-fault, open-field, inclined screen and postural reflex tests, a tape-removal test was the only one that showed effects of an age, treatment and a recovery duration, suggesting that this tape-removal test provides the most tractable measure correlating to the extent of cortical damages following ischemic stroke [46]. In that study, however, the authors did not observe a group difference between control and ischemic animals by rotarod test probably due to technical problems (for examples, some mice were motivated to jump off from the treadmill during tests). Consistent with this report, we also failed to find a correlation between the extent of morphological protection by 29D7 and an increased retention on the treadmill in the rotarod test (Fig. 6C). Many lines of evidence demonstrate that post-injury hypothermia elicits neuroprotection after ischemic stroke in adult rodents [62], [63], [64] and after hypoxia-ischemia in neonatal animals [65], [66]. We found that 29D7 did not affect the body temperature up to 7 days after H-I in P7 rats (Fig. 5), suggesting hypothermia does not appear to account for 29D7’s neuroprotective action.

We found that icv injection of 29D7 increased the TrkB phosphorylation to a same extent in both male and female rats (Fig. 1C). A recent study showed that TrkB activation by a flavonoid analog 7,8 dihydroxyflavone afforded significant neuroprotection in female, but not in male, when hippocampal neuronal damage was assessed 72 h following H-I in P9 neonatal mice [67]. The authors reported that 7,8 dihydroxyflavone increased the TrkB phosphorylation to a greater extent in female mice than in male mice following H-I, which may account for gender-depend neuroprotective action of this compound. Interestingly, H-I insult alone also increased the TrkB phosphorylation almost equally in both genders [67]. In addition to TrkB, 7,8 dihydroxyflavone is shown to have a variety of biochemical properties, including antioxidant activity [68], downregulation of cyclin E [69], and antiinflammation via inhibiting NF-κB activation and MAPK activation [69], which may also contribute, at least in part, to neuroprotective action of this compound. The TrkB-selective agonist 29D7, therefore, would provide as an indispensible tool for further studies to explore wether TrkB activation leads to neuroprotection preferentially in female animals following H-I.

In summary, the present study shows that the TrkB agonist 29D7 provides long-lasting neuroprotection against neonatal H-I injury in vivo. Additional studies with post treatment paradigms should further be able to shed lights on the potential clinical utility of 29D7 in the treatment of H-I brain injury in developing brain.
